# Understanding the Fidelity and Specificity of DNA Polymerase I

**DOI:** 10.1021/acsomega.5c07534

**Published:** 2025-12-15

**Authors:** Bill R. Miller, Andrew V. Yeager, Jake A. Collins, Angus Beane, Alexis Blake, Elise Tate, Carol A. Parish, Eugene Y. Wu

**Affiliations:** † Department of Biochemistry, A.T. Still University, 800 W. Jefferson St, Kirksville, Missouri 63501, United States; ‡ Department of Chemistry, 4271 Truman State University, 100 E. Normal Ave, Kirksville, Missouri 63501, United States; § Department of Chemistry, 6888 University of Richmond, 28 Westhampton Way, Richmond, Virginia 23173, United States; ∥ Department of Biology, University of Richmond, 28 Westhampton Way, Richmond, Virginia 23173, United States

## Abstract

High-fidelity DNA polymerases employ complex mechanisms to catalyze template-dependent DNA synthesis while quickly discarding mismatches. Atomic-level structural details about short-lived states during nucleotide discrimination are necessary to gain insight into the kinetic checkpoints that contribute to fidelity. We performed microsecond molecular dynamics simulations of DNA polymerase I, large fragment, from *Bacillus stearothermophilus* (Bacillus fragment, or BF) in complex with a template guanine and a mismatched thymidine triphosphate to observe the early events in the process of selection against a mismatch. Although the nucleobases formed a wobble base pair early, the mismatched pair was blocked from fully entering the active site by a conserved tyrosine, Tyr714, leading to the eventual disruption of the unstable pair. Simulations of the mutant BF at residue 714 reveal that a serine mutation readily accommodates a G-T mismatch, explaining the results of previous studies. Mismatch G-G simulations reproduce previous DNA polymerase crystal structures and further support the importance of Tyr714 in DNA polymerase fidelity. Our molecular dynamics studies of BF provide strong evidence for a multiconformational, stepwise selection mechanism that disfavors unstable mismatches prior to closure. Our free energy calculations indicate a substantial barrier between the closed and ajar states. This suggests that once the ternary complex fully closes, it will likely remain closed, regardless of whether complementary or noncomplementary nucleotides are present in the active site. Dynamic discrimination against mismatches leads to nucleotide dissociation and contributes to DNA replication fidelity in DNA polymerase I.

## Graphical abstract

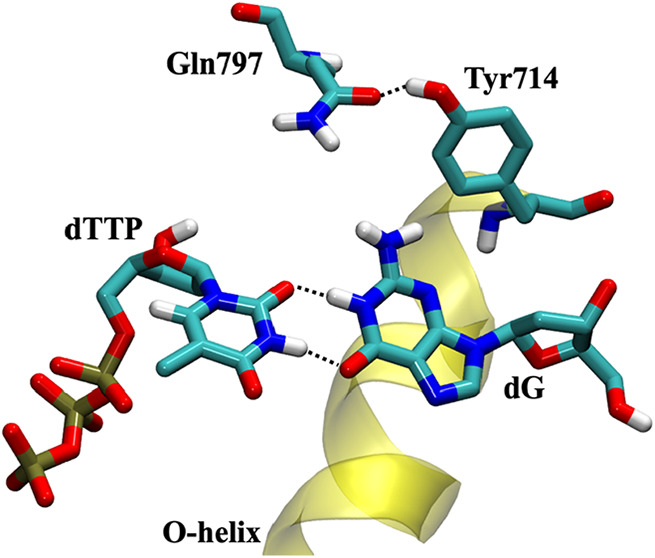

## Introduction

DNA polymerases, which catalyze DNA replication in organisms, quickly distinguish between structurally and chemically similar 2'-deoxynucleoside triphosphates (dNTPs) and ribonucleoside triphosphates (rNTPs) in solution. The enzymes must accurately match a dNTP ligand with its complementary nucleotide on the template DNA strand. Bacterial DNA polymerase I enzymes reach nucleotide incorporation rates of tens to hundreds of nucleotides per second, while making only one error every ∼10^5^ bases.^1^ This high accuracy rate is unexpected if based solely on the small thermodynamic variability between correct and incorrect base pairs.^2–4^ Therefore, the active site of the DNA polymerase must play a key role in the selection and binding of complementary dNTPs that form proper Watson–Crick (WC) base pairs during DNA replication.

Bacterial DNA polymerase I and homologues (termed “A-family” DNA polymerases) generally resemble a human hand, including a “thumb” subdomain that grasps the double-stranded DNA, a “palm” subdomain that includes the active site, and a mobile “fingers’’ region involved in binding of the complementary dNTP.^5,6^ Previous X-ray crystal structures of DNA polymerase I without its dispensable *N*-terminal 5′-to-3′ exonuclease domain (Klenow fragment) have depicted three major conformations of the fingers subdomain for *Geobacillus stearothermophilus* DNA polymerase I (Bacillus Fragment or BF): closed,^5,7^ ajar,^8,9^ and open.^5,10,11^ Fluorescence studies of A-family DNA polymerases show the closed conformation dominates in the presence of a WC base pair, such as G-C (Figure 1A) or A-T (Figure 1C), formed by the dNTP ligand and the template base, but ajar is most prevalent when a noncomplementary dNTP binds, such as G and T (Figure 1E), to the active site, and the open conformation is most frequently sampled in the absence of a dNTP.^12–15^ The ajar (or “partially closed”) structure has been hypothesized as an early checkpoint for distinguishing between WC and non-WC base pairs.^9,12^ The current literature suggests that if the correct dNTP binds to the active site (i.e., forms a WC base pair), the polymerase proceeds from the open to the ajar and continues to the closed conformation; however, if an incorrect dNTP binds, the ternary state (enzyme + DNA + dNTP) is destabilized prior to closure, leading to dNTP dissociation and reopening of the fingers subdomain. Wu and Beese observed the ajar conformation by making one of three active site mutations: Phenylalanine 710 to tyrosine with dG-ddTTP mismatch (F710Y), tyrosine 714 to serine with dG-dTTP mismatch (Y714S), or valine 713 to proline with dG-dCTP base pair (V713P).^9^ The dynamics and transitions between these conformations have been shown to be critical in the incorporation rates of DNA polymerases.^16–18^

**Figure 1:**
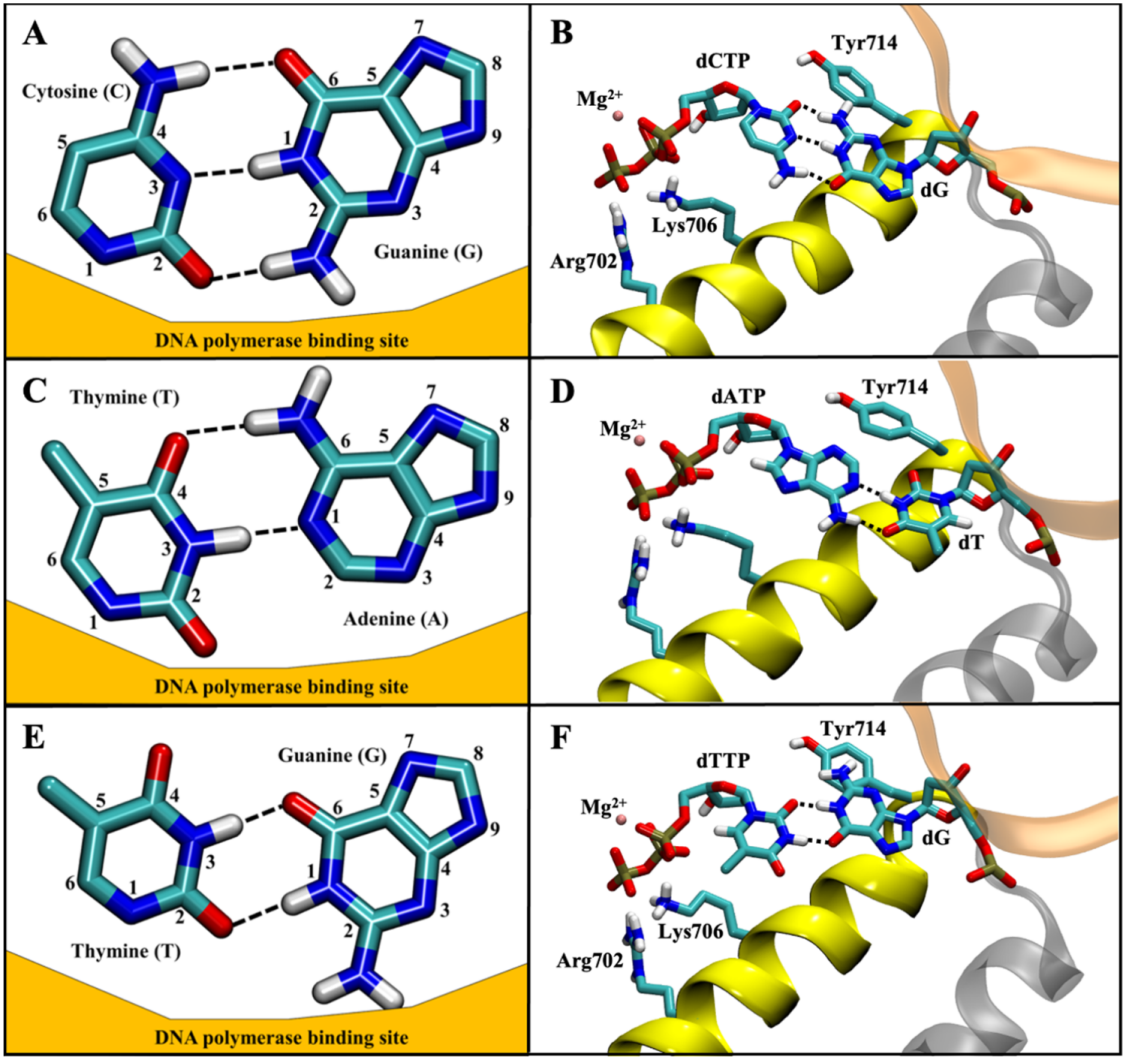
Depiction of three potential base pairs in the DNA polymerase binding site. Dashed black lines indicate hydrogen bonds present between bases. Pairing for a traditional complementary Watson–Crick G-C base pair (A) positioned relative to the surface of the DNA polymerase binding site and (B) within the DNA polymerase binding site displaying key amino acids. Pairing for a traditional complementary Watson–Crick A-T base pair (C) positioned relative to the surface of the DNA polymerase binding site and (D) within the DNA polymerase binding site displaying key amino acids nearby. Pairing between a guanine and thymine that form a wobble G-T base pair (E) positioned relative to the surface of the DNA polymerase binding site and (F) within the DNA polymerase binding site displaying key amino acids, indicating the steric clash between Tyr714 and the G-T base pair.

DNA polymerase fidelity relies on the structural and dynamic selection of nucleotides during and prior to catalysis. Pre-steady-state kinetics experiments on DNA polymerases using excess enzyme over DNA substrate to measure catalytic rates of single nucleotide addition^19–21^ have been shown to be equivalent to steady-state enzyme kinetics for estimating polymerase fidelity^19^ and provide enzyme parameters for nucleotide affinity (dNTP dissociation constant, or *K*_D_) and maximal polymerization rate (*k*_pol_). These kinetic parameters indicate that both dNTP affinity and polymerization rate substantially contribute, sometimes roughly equally, to polymerase fidelity, depending on the polymerase and the nucleotides studied.^18,19,22–24^ Generally, there is a 1–2 orders of magnitude difference between the affinities for the correct and mismatched nucleotide, suggesting that polymerases quickly dissociate from mismatched dNTPs while retaining Watson–Crick base pairs in the active site. Kinetics and crystallography experiments have not yet established in which state polymerases (open, ajar, or closed) dissociate from mismatched nucleotides. The factors and structural features of polymerase active sites that influence the dynamics of mismatched nucleotide dissociation remain unclear.

Recent computational and experimental studies have identified active site residues that are critical for fidelity and preventing misincorporation.^25–27^ In particular, residues most critical for polymerase fidelity form the shape or pocket of the active site that interacts with the incoming dNTP and the base pair within the active site. Kinetic assays previously identified a tyrosine universally conserved in bacterial DNA polymerase I, Tyr714 (using BF numbering), as a key residue in DNA polymerase I fidelity.^28^ Subsequent experiments have suggested that Tyr714 might play a role in positioning and stabilizing the template base^29^ while also aiding the open-to-closed conformational change upon dNTP binding.^14^ Furthermore, crystal structures of DNA polymerase I in the closed conformation show Tyr714 hydrogen bonding with glutamate 658 (Glu658), forming the inside shape of the binding pocket.^5^ In addition to interacting with Tyr714, Glu658 is also implicated in positioning the incoming nucleotide and preventing misincorporation of rNTPs.^11^ Finally, crystal structures show that Phe710 helps arrange the active site through π-stacking with the nucleotide of the dNTP.^5^ Despite the presence of DNA polymerase crystal structures and experimental assays, atomistic details of the dynamics of active site residues during binding of complementary and noncomplementary dNTPs remain unclear. Recent methodological and hardware advances allow computational simulations of proteins and enzymes on the μs time scale to reliably provide this level of detail not available with experimental techniques.^30^ Recent work with DNA pol β and η has demonstrated how molecular dynamics and free energy calculations can be utilized to accurately study the local atomic rearrangements in the active site of DNA polymerase.^31^

Previously, we performed molecular dynamics studies on BF in the presence of a Watson–Crick base pair (dCTP-dG), characterizing the closing process of the enzyme.^32^ More recently, we used molecular dynamics (MD) simulations to investigate the energetics of the relative intermediates in the conformational change from closed to open.^33^ In the present paper, we have performed μs MD simulations of DNA polymerase I in the presence of a mismatch non-Watson–Crick base pair starting from the open (PDB: 4YFU), ajar (PDB: 3HPO), and closed (PDB: 1LV5) conformations. We compare these simulations to our previous results to gain an atomistic understanding of the early events in nucleotide selection by DNA polymerase I. We propose a scheme for nucleotide binding driven by electrostatics between the triphosphate of the dNTP and the active site, followed by polymerase positioning of the ligand opposite the template base. After the base pair forms, active site residues either help stabilize a Watson–Crick base pair (which leads toward catalysis) or destabilize a mismatch (which leads to the nucleobase being ejected from the binding pocket). Additionally, we further elucidate the role of active site residues in dNTP binding and discrimination between correct and incorrect nucleotides. Specifically, we describe the structural role of Tyr714 in destabilizing the dTTP-dG mismatch while contrastingly stabilizing a dTTP-dA base pair. Furthermore, we performed additional simulations of mutant BF at Tyr714 to elucidate the role of residue 714 in the fidelity. Finally, we observed that the dTTP-dG simulation initiated from the closed conformation remains closed during our simulations, suggesting that mismatched nucleotide dissociation does not quickly occur from the closed state, likely due to the observed high free energy barrier between the closed and ajar states.

## Methods

Microsecond time scale classical MD simulations were performed on *G. stearothermophilus* DNA polymerase I starting from the open (PDB: 4YFU), ajar (PDB: 3HPO), and closed (PDB: 1LV5) structures. Prior to simulation, the PDB structures were *in silico* mutated back to the wild-type sequence, reversing mutations used for crystallization. Specifically, V713P and Y714S mutations were needed for crystallization of the ajar conformation,^9^ and a F710Y mutation was needed to produce the open conformation crystal structure.^32^ Additionally, the dNTP in the 1LV5 (closed) PDB was *in silico* mutated to a dTTP (from the original dCTP) to create the dTTP-dG mismatch present in the open and ajar PDBs. For comparison purposes, simulations were performed with a complementary dCTP-dG within the DNA polymerase I active site beginning from each starting conformation (open, ajar, and closed). For further comparison with a more unfavorable mismatch, new systems were also created where each crystal structure was modified to have a dG-dGTP mismatch in the active site. Charges for dNTPs are not provided with the standard Amber force fields; thus, atomic charges for dCTP, dTTP, and dGTP were calculated with HF/6–31G* using Gaussian 09 ^34^ to maintain consistency with the methods used to calculate atomic charges for the standard Amber nucleotide force fields. An example Gaussian 09 input file for dTTP is provided in the SI (Figure S7), along with calculated partial atomic charges for dCTP, dTTP, and dGTP (Figures S8–S10). Initial coordinates for the Gaussian 09 charge calculations were obtained from the dNTPs within the DNA polymerase I active site from their respective PDBs: dCTP from 1LV5, dTTP from PDB 3HPO, and dGTP was generated by adding two phosphate groups to a dGMP (DG36) in 1LV5. The Amber *ff12SB* force field^38^ was used for the remaining dNTP parameters (e.g., bonds, angles, dihedrals, etc.). Magnesium­(II) ions were placed in metal site B (coordinated to the triphosphate) if necessary. Magnesium ion parameters were obtained from Allnér et al.^35^ Each structure was neutralized with sodium ions and simulated using periodic boundary conditions in a truncated octahedron filled with TIP3P explicit water molecules^36^ to allow for at least 12.0 Å of distance between the protein and the unit cell sides, for a total of ∼80,000 atoms per unit cell (Figure S11).

All trajectories were simulated using the GPU-accelerated version of the Amber MD software package^37^ and the Amber *ff12SB* force field^38^ at constant temperature (335 K) and pressure (1 atm) using the same procedures and variables as previously described for the closing process.^32,33^ All initial structures underwent a seven-stage minimization procedure (Figure S12) involving a maximum of 1000 steps of steepest descent minimization, followed by up to 4000 steps of conjugate gradient minimization at each stage. Positional restraints on all heavy atoms were initially set to 10.0 kcal/mol/Å^2^ and gradually lowered during each stage to zero for the final (seventh) stage. After minimization, each system was heated linearly from 10 to 335 K over 2.0 ns, while positional restraints on heavy atoms were held constant at 10.0 kcal/mol/Å^2^ on the DNA–protein complex (Figure S13). Finally, each system was equilibrated by running MD at constant temperature (335 K) for 3.5 ns and systematically lowering the restraints at each 0.5 ns internal until reaching a final restraint weight of zero (unrestrained) over the final 0.5 ns (Figure S14). Unrestrained MD (Figure S15) was performed on all systems at constant pressure (1 atm) using a Berendsen thermostat with isotropic positioning and constant temperature (335 K) with a Langevin thermostat^39^ using periodic boundary conditions, saving the coordinates, velocities, and energies every 100 ps. Long-range interactions were treated with the particle mesh Ewald method for periodic boundaries using a nonbonded cutoff of 9.0 Å, and the nonbonded atom list was updated every 25 steps (default). New random number seeds were chosen every 25 ns for each simulation to prevent synchronization of the trajectories.^40^ The SHAKE algorithm^41^ was used to fix all covalent bonds involving hydrogen atoms, allowing a 2.0 fs time-step for dynamics. All simulations were performed for at least 3.5 μs of unrestrained MD (Table S1).

Simulations were visualized and analyzed using VMD^42^ and *cpptraj*,^43^ respectively. Molecular mechanics-generalized born surface area (MM-GBSA) free energy calculations were performed using the *MMPBSA.py* program^43^ in Amber and excluded all direct entropic contributions. MM-GBSA free energies were calculated every 0.1 ns (all saved frames) using an implicit GB solvent model (igb = 2) in conjunction with *bondi* radii parameters. All other parameters for *MMPBSA.py* were set to default values (Figure S16).

## Results and Discussion

### Simulation Stability

Simulations were analyzed for structural stability by using root-mean-square deviation (RMSD) and per-residue root-mean-square fluctuations (RMSf). These results suggest that the structures are all relatively stable; the average RMSD for each simulation was less than 3.5 Å (Figure S1). As expected, the structures beginning from the closed conformation (average RMSD: 2.6 Å) were less dynamic than simulations starting from the ajar (average RMSD: 2.8 Å) and open (average RMSD: 3.1 Å) conformations, regardless of the base pair in the active site. Per-residue RMSf results (Figure S2) were consistent with the RMSD data, suggesting the dynamics of the DNA polymerase in order from least to most were simulations starting from the closed, ajar, and open conformations, respectively.

### Scheme for Nucleotide Binding and Selection by DNA Polymerase I

We computationally simulated DNA polymerase I in the presence of a dTTP-dG mismatch in the active site starting from the open, ajar, and closed conformations. We compared these simulations to results from our previous study with a complementary dCTP-dG base pair in the active site^32^ to propose a dNTP selection scheme for DNA polymerase I (Figure 2). Regardless of the identity of the incoming dNTP, Step 1 of dNTP binding involves the long-range Coulombic attraction between the negatively charged triphosphate of the ligand and positively charged amino acids in the polymerase active site (Arg702, His682, and Lys706). Next, the dNTP base is guided to a position near the active site through a π-stacking interaction with Phe710 on the O-helix. This new position allows the dNTP base to form hydrogen bonds with the base on the template strand, as shown in Step 3 of Figure 2. Steps 1–3 are the same regardless of the incoming dNTP; however, the following steps differ depending on the ability of the dNTP to form a Watson–Crick base pair with the template base.

**Figure 2:**
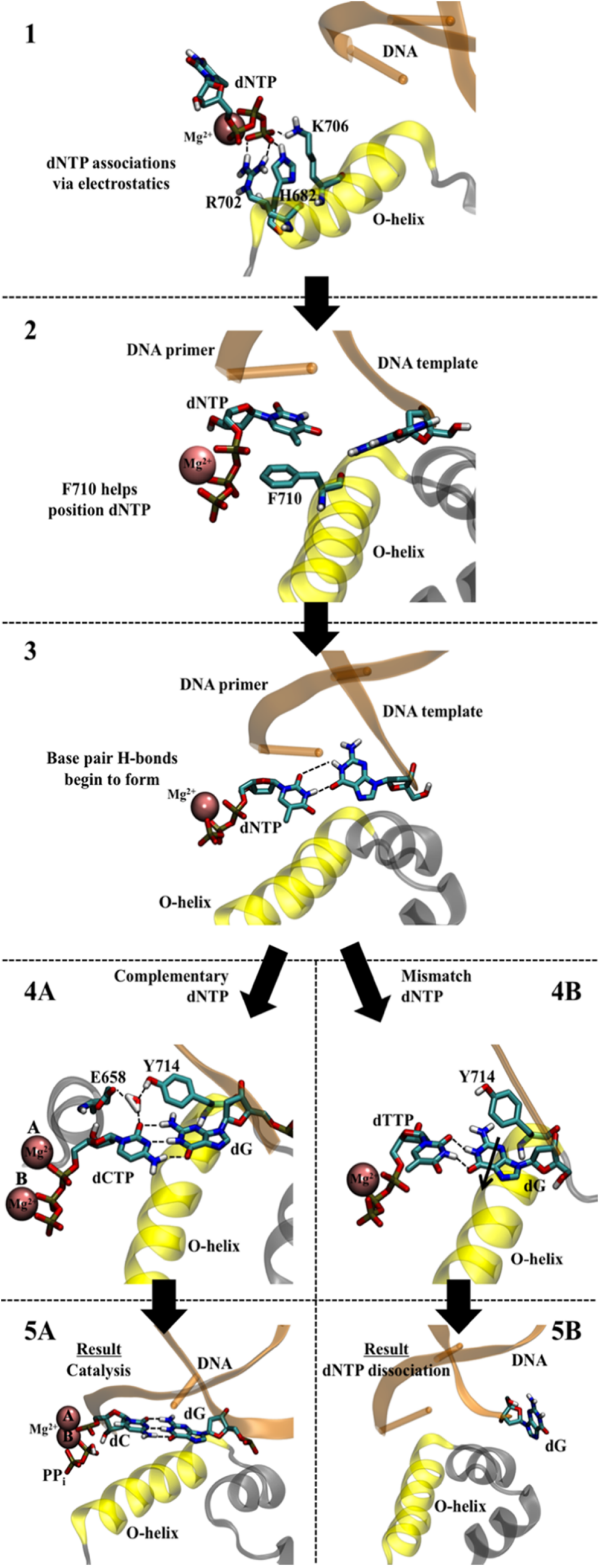
Proposed scheme for dNTP selection by DNA polymerase I. Steps 1–3 occur regardless of the particular dNTP. Steps 4 and 5 are unique as a complementary dNTP (shown as a dCTP opposite a dG) leads to closure of the active site with Mg^2+^ ions present in metal sites A and B (followed by catalysis, 5A), while a mismatch dNTP (shown as a dTTP opposite a dG) leads to the active site opening and dissociation of the incorrect dNTP (5B).

If the incoming dNTP binds to form a WC base pair, then the binding process proceeds through Steps 4A and 5A in Figure 2. We previously observed the binding process from simulations with a dCTP-dG base pair in the polymerase active site.^32^ Initially, a magnesium­(II) ion binds to metal site A helping promote the closure of the fingers subdomain. A hydrogen bonding network between Glu658, Tyr714, and the base pairs promotes a stable conformation in the active site. Depending on the nature of the base pair, a water molecule may also be present in the hydrogen bonding network. The polymerase progresses to full closure of the active site and results in catalysis where the nucleotide from the dNTP is added to the 3′-end of the primer strand (Figure 2 Step 5A).

### Discrimination of a dTTP Opposite a Template dG Base

To visualize nucleotide discrimination in the polymerase active site, we performed microsecond-scale, explicit solvent molecular dynamics simulations of BF bound to dTTP mismatched to a template 2′-deoxyguanosine template. We chose to study the dTTP-dG mismatch because of the presence of high-resolution crystal structures of BF ternary complexes with this mismatch.^9,32^ With the complementary dCTP-dG base pair, we observed that the polymerase recognizes and closes around the active site. In contrast, we expected DNA polymerase to recognize dTTP as an incorrect match opposite the template dG, leading to dissociation from the active site. With the exception of the simulation initiated from the closed conformation (discussed in more detail below), the simulations resulted in the nucleobase dissociating from the polymerase active site driving the fingers subdomain into the open conformation. However, it should be noted that the dTTP never fully dissociated from the polymerase as the triphosphate remained bound through strong electrostatic interactions with the N and O helices on the polymerase. The fingers subdomain was destabilized quickly, resulting in the opening of the fingers domain after <50 ns (Figure 3) for the simulation started from the ajar conformation, and even faster for the simulation began using the open conformation. In contrast, the simulation started from the closed conformation with a mismatched dTTP opposite dG remained closed for the duration of the simulation (Figures 3, 4, and S3).

**Figure 3:**
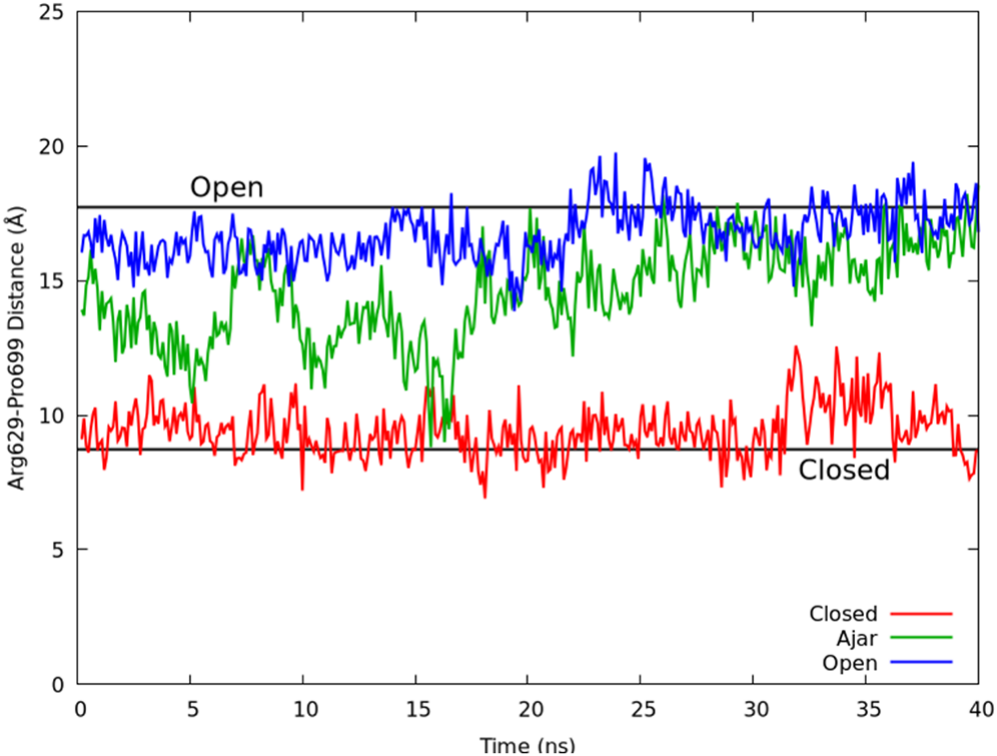
Opening of the fingers domain for the dTTP-dG mismatch simulations initiated from the open (PDB 4YFU, blue), ajar (PDB 3HPO, green), and closed (PDB 1LV5, red) conformations. The distance for the crystallographic open distance (17.5 Å) is shown for reference as a solid black line. The opening is measured using the distance between the relatively stationary Arg629 in the palm domain and the more mobile Pro699 found in the O-helix of the fingers domain. Note that only the first 40 ns of the 1.0 μs simulations is displayed to depict the opening process at the beginning of the simulation starting from the ajar conformation. The full-length distances are shown in Figure S3.

**Figure 4:**
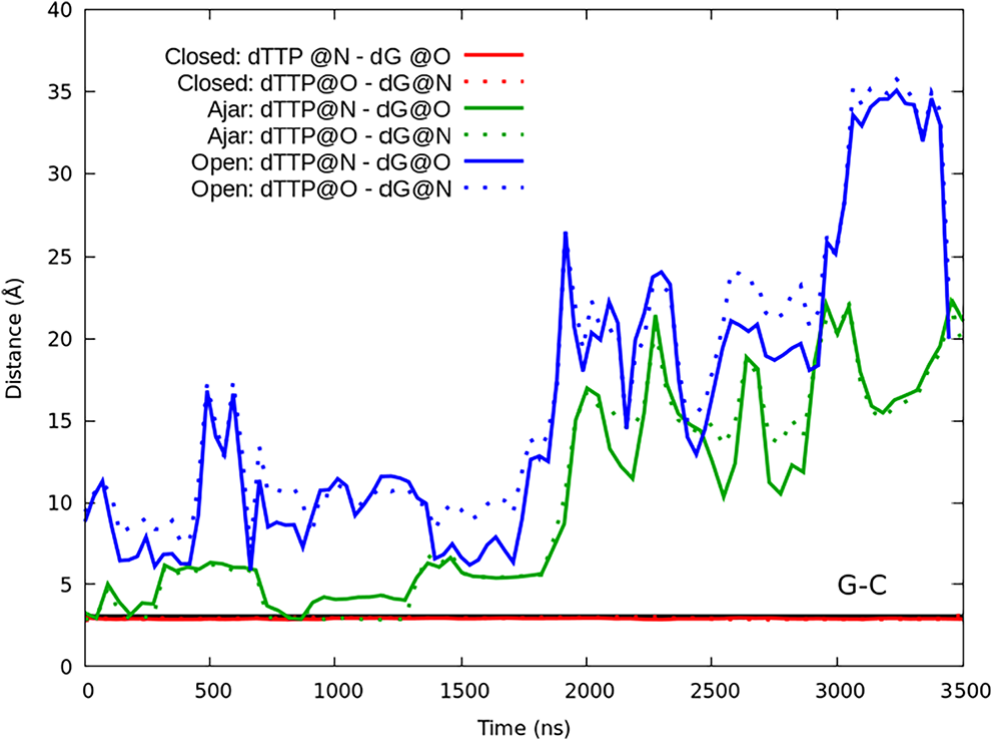
Distances (as running averages) between potential hydrogen bond partners for the dTTP and dG bases (see Figure 1E for reference) from the simulations initiated from the closed (PDB 1LV5, red), ajar (PDB 3HPO, green), and open (PDB 4YFU, blue) crystal structures. For reference, the distance between hydrogen bond partners for a typical dG-dC base pair is shown at ∼3 Å.

The opening of the fingers subdomain exposes the active site to the solvent, aiding the dissociation of the improper nucleobase and preventing misincorporation by the enzyme. In the open crystal structure (PDB 4YFU), the dTTP and template dG nucleotides are not paired as expected from a wobble T-G base pair (i.e., the bases are not in close enough proximity or orientation to hydrogen bond with one another). However, during the simulation initiated from the open conformation, the two bases contacted one another several times over the first ∼1.8 μs before the nucleobase eventually dissociated from the active site at ∼1.9 μs (Figure 4).

From the ajar crystal structure (PDB entry3HPO), the dTTP and template dG nucleotides form a wobble T-G base pair (Figure 1E). Despite the presence of this wobble base pair, the dTTP-dG interaction is not stable. Early in the simulation, the base pair becomes destabilized, and the hydrogen bonding between the dTTP and dG bases is severed when the template dG base leaves the active site at ∼1900 ns (Figure 4). In both the open and ajar simulations, despite repeated and extensive contact between hydrogen bonding partners, the mismatched dNTP does not drive the polymerase toward closure.

At the atomic level, we propose that the nucleotide selection process following Step 3 predominantly takes an alternate pathway illustrated by Steps 4B and 5B upon binding a mismatched nucleotide (shown with a dTTP-dG mismatch in Figure 2). Any hydrogen bonding between the mismatched base pairs leads to clashes with the active site residues in the polymerase. In the dTTP-dG mismatch example shown in Figure 2, the wobble base pair attempts to fit into the active site, but this movement forces the amino group on the dG base to clash with the aromatic side chain of Tyr714 (Step 4B in Figure 2). This is consistent with studies stating that the shape of the active site near the Tyr714-Glu658 interface was critical to active site specificity.^32,44,45^ In our simulation, the tyrosine does not vacate its location, which blocks the dG base from the active site along with the dTTP substrate. Without the stability provided by the Glu658 and Tyr714 active site residues, the base pair is unable to maintain its hydrogen bonding interactions, leading to complete dissociation of the dTTP from the active site and opening of the polymerase to allow binding of another dNTP in solution.

### Free Energy Barrier Analysis of WC and Non-WC Base Pairs

Determination of the free energy landscape associated with the fidelity of DNA polymerase is critical to our understanding of the enzyme’s ability to discriminate between the correct and incorrect ligands.^18,27^ Computational methods were previously utilized to investigate the free energies associated with the fidelity of similar DNA polymerases.^46,47^ Our extensive simulation time on BF starting from the open, ajar, and closed conformations in the presence of both WC base pairs and non-WC base pairs in the active site has allowed us to describe an estimation of the relative free energy landscape for the early stages of the prechemistry steps in nucleotide selection for BF DNA polymerase I (Figure 5). To generate this schematic, we performed molecular mechanics-generalized born surface area (MM-GBSA) free energy calculations on all conformations of DNA polymerase observed in our mismatch (dTTP-dG pair) simulations described herein, along with our previously published ternary simulations (dCTP-dG).^32^ The MM-GBSA free energy value for each frame was mapped with the corresponding nucleophilic attack distance (i.e., distance between the 3′–OH and the dNTP α-phosphate) for each simulation and then organized into bins based on the distances using an in-house Python script to generate Figure 5. Our MM-GBSA free energy estimates should not be considered absolute, but their relative energies have often been used to compare the stability of structures in the literature.^48^ We recognize that the magnitudes of the energies presented are misleading (likely caused by the number of solute atoms involved in the calculations not being localized to the active site), but instead, we are mostly concerned with the observed trends in the free energy estimates as the polymerase changes conformations. The relative shape of the free energy estimations determined herein is consistent with that described experimentally using FRET by Hohlbein et al.,^14^ with few exceptions. Additionally, our free energy profiles (Figure 5) are qualitatively consistent with results described by Kirmizialtin et al. on HIV reverse transcriptase in the presence of a nucleotide match and mismatch.^47^ Three conformations (closed, ajar, and open) have been resolved experimentally,^5,9,32^ and with our simulations, we expected to see at least three distinct minima in the free energy estimation graphs that are in line with previous findings. Using multiple metrics, our simulations suggest three distinct, energetically stable conformations of DNA polymerase I. The distance between four residues in the polymerase (R634, R629, I636, and W872) and the Pro699 residue on the mobile O-helix was measured and compared with the nucleophilic attack distance between the 3′–OH and the dNTP α-phosphate (Figure S4). The mobile O-helix was used as it is traditionally a major indicator of polymerase conformation.^9,11,49,50^ These four residues were chosen because they represent four parts of the protein core that are considered generally stationary, and as such, any distance changes reflect movement of the O-helix. Based on our use of multiple metrics, the nucleophilic attack distance appears to give the most accurate representation of protein conformation (Figure 5). Previous literature has suggested that the rate-limiting step for DNA polymerase I may be a conformational change rather than a chemical reaction.^51–55^ We propose, based on our free energy estimations, that the conformational change with the highest energy barrier in the closing mechanism is the conformational change from ajar to closed. In forming this conclusion, we considered the closed structure to maintain the shortest nucleophilic attack distances (3–5 Å), while the ajar and open conformations’ sample distances of 8–10 and 11–12 Å, respectively. Once the polymerase closes around the base pair, it becomes energetically unfavorable to transition back to the ajar conformation due to the steep drop off in free energy associated with the ajar-to-closed change. The energy barrier estimations associated with the G-C WC base pair and the G-T non-WC are very similar to each other, likely due to G-T being a more commonly inserted mismatch and the purine-pyrimidine pairing being more favorable (Figure 5). The rate-limiting step for nucleotide insertion by DNA polymerase has been a contested point in the literature,^56^ but our simulations suggest that among the prechemistry steps involved, the slowest (highest energy barrier) step appears to be closure of the active site from the ajar conformation. In particular, during this study and previous studies,^33,57^ we have not observed DNA polymerase transition from the open or ajar state to the fully closed conformation, even in the presence of WC base pairs, likely due to the limited simulation time relative to biological time frames. Additionally, and potentially more supportive of this hypothesis, we do not observe the DNA polymerase transition from the closed to open conformation in any simulations, including all mismatch base pairs. If the mismatch simulations were always unstable in the polymerase binding pocket, then we would expect to observe the opening of the O-helix and the dissociation of the incorrect dNTP. Instead, our simulations suggest that once the active site has overcome the large energetic barrier of reorganizing the residues in the binding pocket to accommodate the base pair (mismatch or otherwise), it becomes increasingly difficult to release the dNTP. Each of our mismatch simulations depicts an active site with stabilizing intermolecular interactions and minimal large-scale movement of the polymerase backbone that would be necessary to allow opening of the binding pocket under these time scales. Our previous work^33^ indicates that the closed conformation is energetically more stable than the ajar or open conformations for nearly every possible base pair combination. The data herein supports the previous work^33^ that once the polymerase closes around a dNTP, regardless of the identity as a WC or non-WC pair, it becomes energetically more stable. The calculated MM-GBSA relative free energy estimate barrier for transitioning from closed to ajar is ∼900 kcal/mol, though as previously noted, we acknowledge that the limitations of the simplistic MM-GBSA free energy calculation method have led to an unnaturally stable energy for the closed conformation. However, this study supports the hypothesis that the transition of the DNA polymerase active site from ajar to closed is the slowest of the prechemistry steps for nucleotide insertion. Our free energy estimates for the non-WC base pair G-G are substantially higher (by ∼75 kcal/mol), suggesting that it is much more difficult to bind a purine–purine mismatch in the active site. These simulations support the hypothesis that the energy barriers play a large role in the selection of correct base pair incorporation.

**Figure 5:**
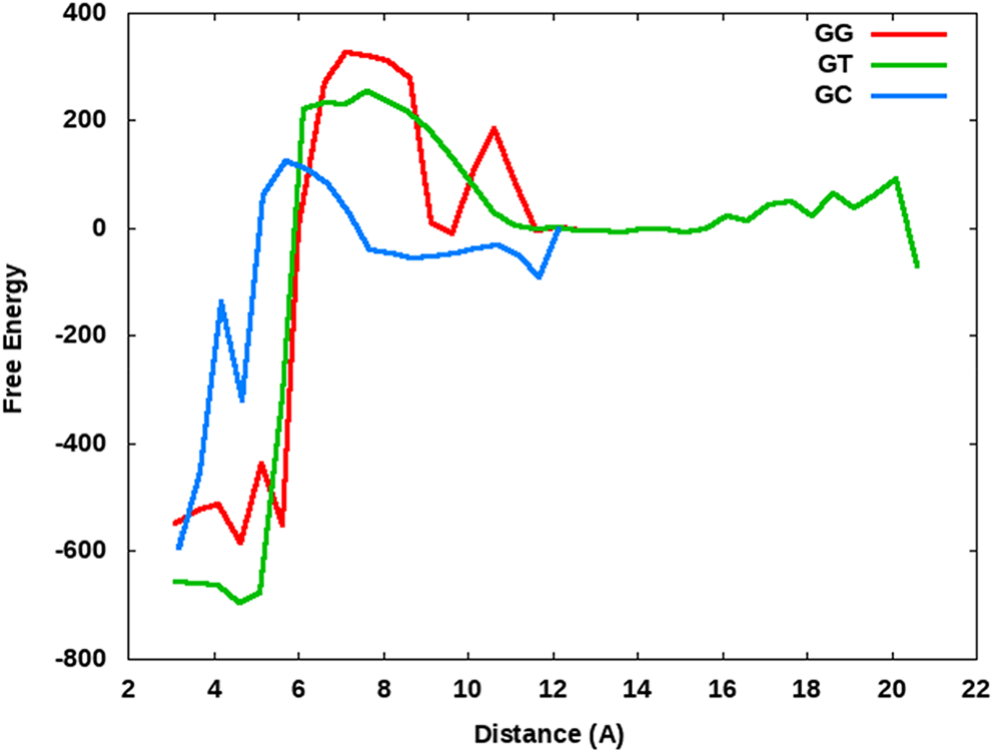
Relative free energy estimate landscape for the prechemistry events for DNA polymerase I in the case of a complementary base pair (G-C) and two mismatched, noncomplementary base pairs (G-T and G-G). The distance shown corresponds to the nucleophilic attack distance between the 3′–OH and the dNTP α-phosphate. A comparison of multiple analysis metrics suggests that the nucleophilic attack distance is the most accurate gauge of polymerase conformation. Generally, this distance shows three distinct, stable conformations of DNA polymerase I corresponding to closed (3–5 Å), ajar (8–10 Å) and open (11–12 Å) structures. Minima corresponding to these three polymerase conformations can be seen most clearly in the GG (red) and GC (blue) simulations, while for the GT (green) simulations, the ajar and open conformations share a broad and relatively shallow basin. Free energy estimates are shown in kcal/mol and do not directly include entropic contributions.

### Role of Tyr714 in GT Mismatch Discrimination

BF was previously resolved by Wu and Beese with a Y714S mutation to accommodate the G-T mismatch present in the active site.^9^ We further investigated the structural and dynamic significance of this mutation with respect to the DNA polymerase binding site. In contrast to the G-T mismatch simulation with wild-type BF, the Y714S mutant BF simulations suggest that the serine stabilizes the G-T mismatch through a persistent hydrogen bond between the side chain hydroxyl on Ser714 and the −NH_2_ group on the template dG (Figure 6A). This hydrogen bond was present for 92% of the Y714S mutant MD simulation, indicating its importance to the local stability of these residues. Additionally, we observed the nucleophilic attack distance to remain short for the G-T mismatch with the Y714S mutant, while the wild type displayed a significantly larger distance between the catalytic atoms, further suggesting that the Y714S mutation allows the G-T mismatch in the active site to be stabilized in a more closed conformation (Figure S5). The Y714S mutation clearly stabilizes this dTTP-dG mismatch; however, we would not expect this mutation to universally stabilize mismatch base pairs within the DNA polymerase active site because the same mutation in DNA polymerase I from *Escherichia coli*, Klenow fragment, shows a clear preference for the dTTP-dG mismatch.^29,58^ Ser714 has a specific hydrogen binding interaction with the template guanine *and* helps position the base to make two favorable hydrogen bonds with the corresponding dTTP ligand. Both of these are unlikely to occur in the presence of a different mismatch.

**Figure 6:**
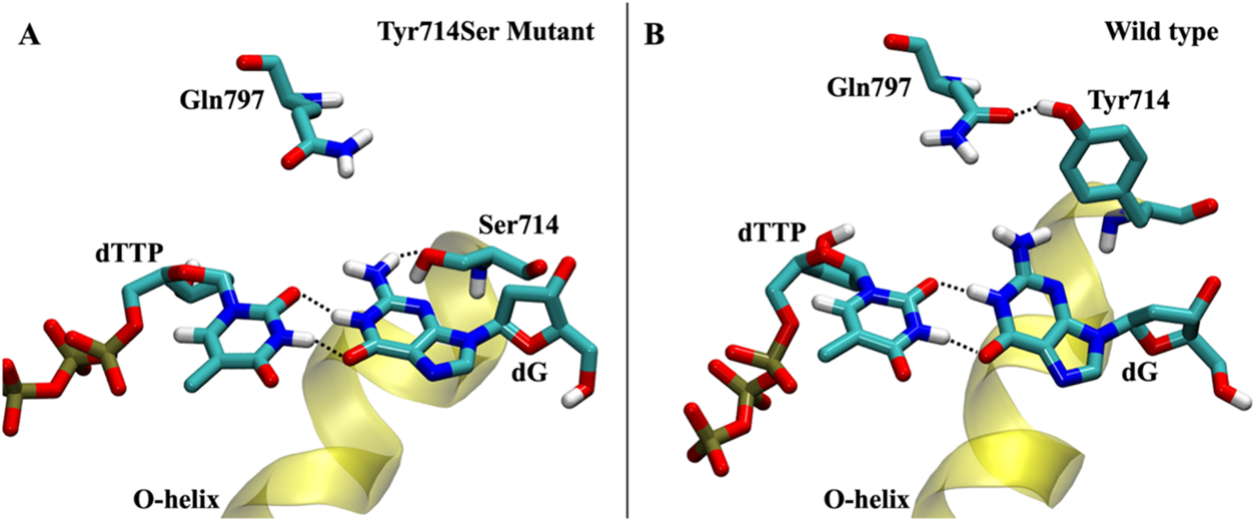
DNA polymerase I active site comparison between the wild type and mutant. (A) Mismatch G-T base pair is stabilized in the presence of the mutant Ser714 due to a persistent hydrogen bond between Ser714 at the -NH_2_ on the dG base. (B) Wild type destabilizes the G-T mismatch due to the bulky Tyr714 creating a steric clash and twist of the template guanine that eventually results in the breaking of the two hydrogen bonds holding the mismatch base pair together.

The smaller size of the serine side chain allows the dTTP-dG mismatch to insert further into the active site than would be allowed by the bulkier tyrosine side chain present in the wild-type structure. In fact, wild-type simulations depict the role of Tyr714 in DNA polymerase specificity (Figure 6B), including the relatively stable G-T mismatch scenario. In particular, Tyr714 hydrogen bonds to the side chain of Gln797, forming the interior of the DNA polymerase binding pocket. Unlike the Y714S mutant, tyrosine prevents the G-T mismatch from inserting deep into the pocket. Instead, the Tyr714 side chain structurally competes for the same space as the template guanine, forcing the guanine to rotate around the glycosidic bond. The rotation of the guanine breaks the planarity of the G-T base pair, eventually breaking the hydrogen bonds between the guanine and thymine since the thymine cannot also rotate due to structural constraints created by base stacking of the dNTP with the n_–1_ nucleotide on the growing strand and Phe710. Tyr714 forces dG out of the binding pocket of the DNA polymerase, which then facilitates dTTP dissociation from the active site. The binding pocket then becomes open and available for binding of a subsequent dNTP. Despite Tyr714 not directly interacting with the incorrect dNTP, the mismatch is unstable in the DNA polymerase active site as a direct result of the formation of the binding pocket surface by Tyr714.

Our simulations suggest that Tyr714 has an indirect role in discrimination of dNTPs within the DNA polymerase active site, since it has no specific interactions with the base pair itself. Instead, our results suggest that Tyr714 controls the volume of the binding pocket, preventing improper base pairs from fully inserting themselves into the active site and thus preventing catalysis. These findings are consistent with *in vitro* experiments describing the important, yet nonspecific role of this conserved tyrosine in DNA polymerase specificity.^44,58^ We anticipate that Tyr714 behaves similarly for alternative mismatch simulations that reach the ajar conformation.

### Discrimination of a dGTP Opposite a Template dG Base

To contrast with a relatively stable G-T mismatch, we also chose to simulate a more disordered G-G mismatch starting from all three starting conformations (closed, ajar, and open). We observed only the dGTP ligand displaced from the active site in the simulation begun from the ajar simulation. An interesting observation across our G-G simulations was the ability of the dGTP to rotate from the *anti-* to the *syn-*conformation within the polymerase active site. When the binding pocket had ample room (such as for the simulations begun in the ajar and open conformations), we observed that the dGTP spontaneously flipped from *anti*- to *syn-* after a few hundred ns (Figure S6). The positioning of Phe710 was critical to allowing dGTP to rotate within the crowded binding pocket. Normally, Phe710 helps position the aromatic nitrogenous base through π-stacking, but small displacements of Phe710 away from the base allow enough room for the base to flip 180° to form the more structurally stable “flipped” dGTP-dG mismatch. The entire dGTP was also observed to rotate (not only the base) within the active site, allowing the dGTP to hydrogen bond with the template dG, and further stabilized by the catalytic Mg^2+^ (or Glu658) coordinating to the dGTP 3′–OH instead of the P_α_ group. Experimental structures support this more stable form of the dGTP-G mismatch with DNA polymerase.^59^ Based on these results, we also simulated the *syn-*dGTP-G in the closed conformation and saw increased stability relative to the *anti-*G-G closed simulation.

Several amino acids are critical to base pair discrimination in the presence of a dGTP-dG mismatch. In particular, Tyr714 and Glu658 are observed to be hydrogen bonding to form the back surface of the active site. The dGTP ligand was observed to dissociate from the active site starting from the ajar conformation, as expected. The mechanism for this mismatch discrimination was unique compared to that of the dTTP-dG simulations (described above). Early in the simulation, Tyr714 replaces the template dG in the active site, pushing the template base back into the preinsertion site. Tyr714 then forms a hydrogen bond with dGTP (Figure 7A). After ∼600 ns, Phe710 rotates into the active site, pushing the dGTP base out of the binding pocket (Figure 7B), effectively removing it from consideration as a potential match for the template base. It is interesting to note the differences in ligand discrimination between dTTP-dG and dGTP-dG mismatches within the DNA polymerase active site. Although the important amino acids (such as Tyr714) remain constant throughout the simulations, the mechanism for discrimination is different for the two mismatches. Although preliminary, we hypothesize that each possible mismatch may have a unique mechanism for recognizing and removing mismatched dNTPs from the polymerase active site. Future studies will look into this in more detail for the other mismatches.

**Figure 7:**
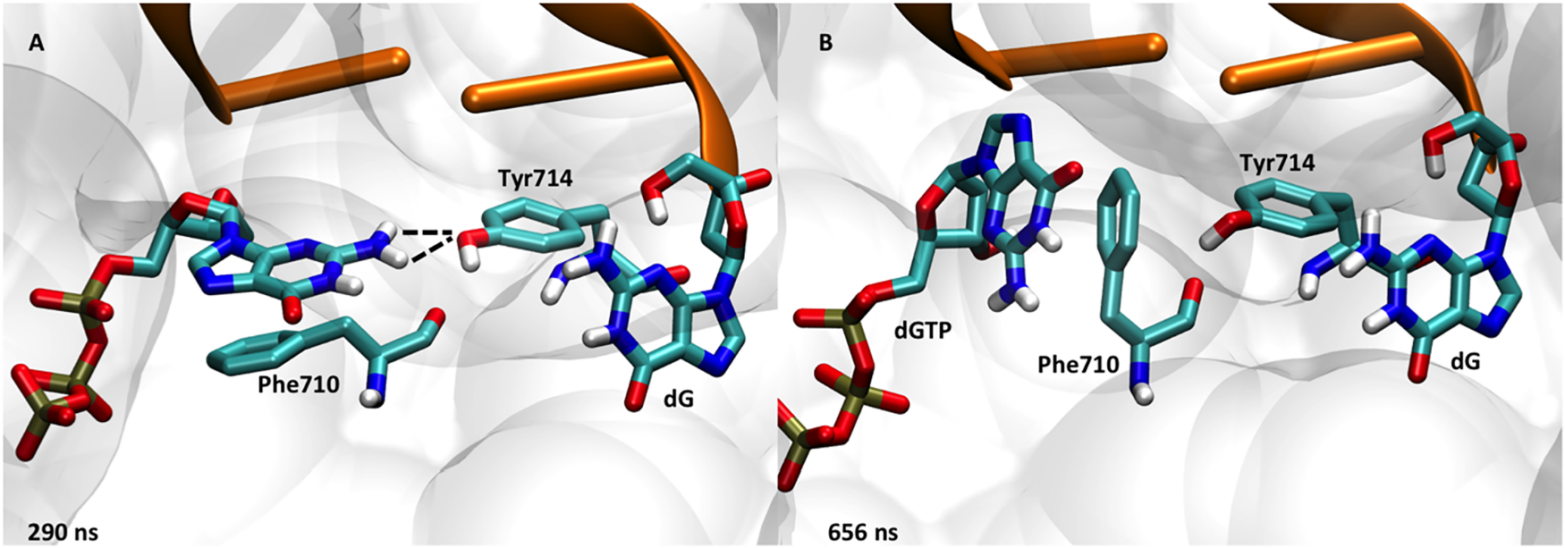
Dissociation mechanism for dGTP-dG mismatch involving (A) Tyr714 initially replacing the template dG in the active site, followed by (B) Phe710 ejecting the base of dGTP from the binding pocket of DNA polymerase.

In our previous work,^32^ we observed a large flip of a conserved histidine (829 in BF) in the palm domain that places the imidazole near the 3′-hydroxyl of the primer and the catalytic metal A site during the MD simulation of wild-type BF, suggesting that the histidine might aid in deprotonating the hydroxyl group for catalysis. The histidine’s backbone torsion angles lie in a disfavored region of the Ramachandran plot in DNA polymerase I crystal structures.^60^ Unlike our previous simulations, both the structural and catalytic magnesium ions were present in the simulations for the current study. In multiple simulations, we observed that the side chain of His829 rotates from its initial conformation in the crystal structure to a state near the active site. When it rotated, we observed His829 near the catalytic Mg^2+^ and hydrogen bonding with the side chain of Tyr714. Furthermore, we observed it to be most structurally stable in the rotated conformation when DNA polymerase was in the closed conformation. Its stability within the closed conformation provides further evidence that His829 may play a role in the removal of the proton from the 3′–OH on the terminal end of the primer strand.^61^ Future studies will utilize QM/MM methods to investigate the potential electronic pathway of 3′–OH proton transfer.

## Conclusions

Simulations of mismatched base pairs (G-T and G-G) in the active site of DNA polymerase provide the first dynamic atomistic details for the high specificity of DNA polymerase. We propose a scheme (Figure 2) for dNTP selection by DNA polymerase I involving the attraction of the negatively charged triphosphate tail to the positively charged binding pocket, followed by the formation of hydrogen bonds between bases. The ∼2 order of magnitude lower affinity for mismatched nucleotides is explained in our simulations by the rapid ejection of unstable partners in hydrogen bonding and/or their position in the active site. Generally, our simulation results are conceptually consistent with a thermodynamic model from homologous T7 DNA polymerase,^18^ wherein the off rate for mismatched nucleotides is much faster than for correct nucleotides, which become trapped in the closed conformation. Furthermore, we detail the critical role of Tyr714 in ejecting improper dNTP ligands from the polymerase binding pocket by using nonspecific interactions with the base pair. This scheme is supported by a comparison of the stability of the wild type with the Y714S mutant used to crystallize the G-T mismatch in the ajar conformation, as well as previous experimental *in vitro* studies.

These MD simulations also provide further support for the ajar-to-closed transition of the DNA polymerase structure as a major thermodynamic barrier for mismatched nucleotides and potentially for nucleotide insertion. Future studies should attempt to directly compare the energetics of the prechemistry events with the catalytic mechanism using a combination of umbrella sampling and mixed quantum mechanics and molecular mechanics (QM/MM). The results of that study would provide a significant discovery in the study of the activity and specificity of DNA polymerase.

## Supplementary Material



## Notes

Table of MD simulation lengths, trajectory RMSD, RMSF and interatomic distance plots, free energy plots, and an atomistic depiction of the conformational flexibility of the dGTP base in the dG-dGTP simulation (PDF)
