# Supplementary material for: Understanding the Fidelity and Specificity of DNA Polymerase I
Source: ACS Omega. 2025 Dec 15;10(51):62746–56. doi: 10.1021/acsomega.5c07534 (PMC12756729; doi:10.1021/acsomega.5c07534)
Supplement: Supplementary file 1 [file ao5c07534_si_001.pdf]

SUPPLEMENTAL INFORMATION

Understanding the fidelity and specificity of DNA polymerase I

Bill R. Miller III[1,2,*], Andrew V. Yeager[2], Jake A. Collins[2], Angus Beane[3], Alexis Blake[3], Elise Tate[3], Carol A. Parish[3,*] and Eugene Y. Wu[4,*]

[1] Department of Biochemistry, A.T. Still University, 800 W. Jefferson St, Kirksville, MO, 63501, USA

[2] Department of Chemistry, Truman State University, 100 E. Normal Ave, Kirksville, MO, 63501, USA

[3] Department of Chemistry, University of Richmond, 28 Westhampton Way, Richmond, VA 23173, USA

[4] Department of Biology, University of Richmond, 28 Westhampton Way, Richmond, VA 23173, USA

* To whom correspondence should be addressed. Tel: (660) 626-2347; Fax: (660) 626-2981; Email: billmiller@atsu.edu. Correspondence may also be addressed to Carol Parish. Tel: (804) 484-1548; Fax: (804) 287-1897; Email: cparish@richmond.edu. Correspondence may also be addressed to Eugene Wu. Tel: (804) 287-6449; Fax: (804) 289-8233; Email: ewu@richmond.edu.

TABLES

Base pair	Conformation	PDB	Simulation time (µs)
dCTP-dG	Closed	1LV5	3.7
	Ajar	3HT3	3.1
	Open	4YFU	4.4
dTTP-dG	Closed	1LV5	6.2
	Ajar	3HPO	4.5
	Open	4YFU	3.5
dGTP-dG	Closed	1LV5	3.8
	Ajar	3HPO	3.7
	Open	4YFU	3.9

Table S1. MD simulation lengths for all nine MD simulations performed on DNA polymerase I starting from the closed, ajar, and open conformations with each of three possible base pairs (dCTP-dG, dTTP-dG, dGTP-dG) within the active site.

FIGURES

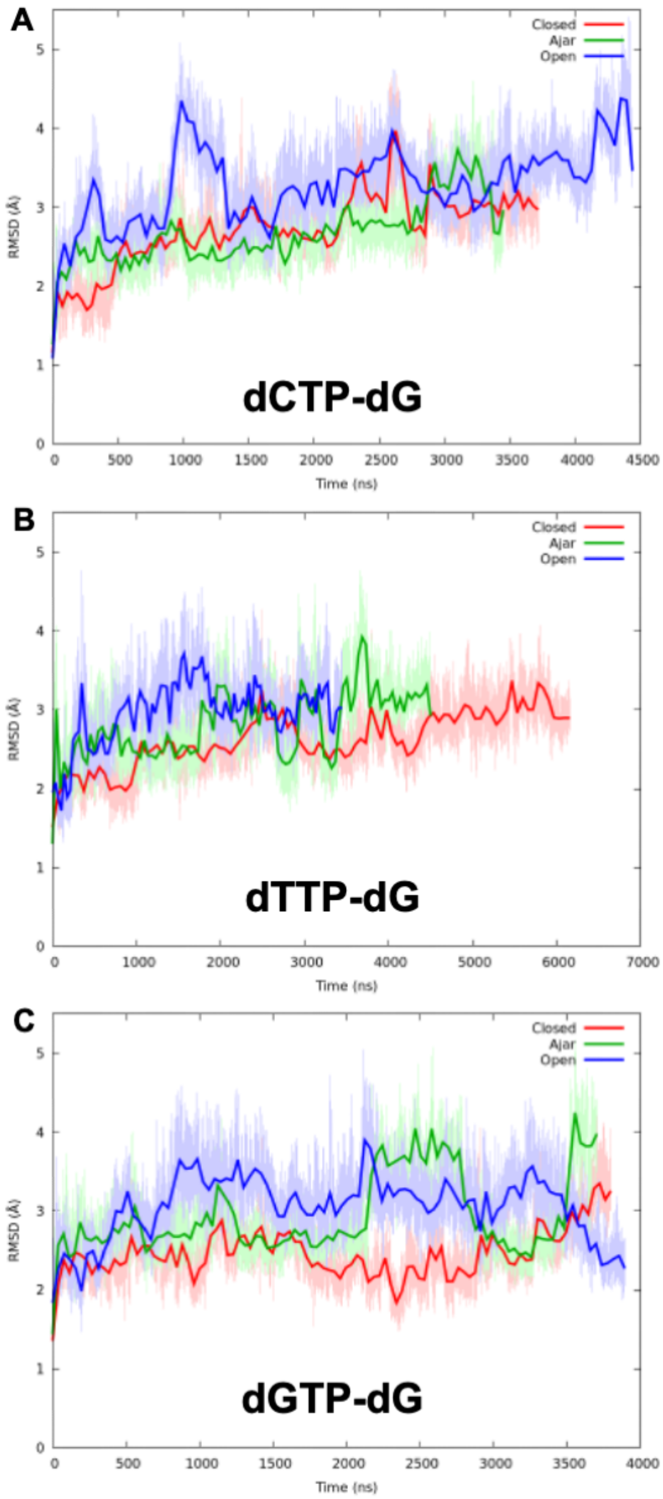

Figure S1. Root mean square deviation (RMSD) plots for the backbone atoms using the corresponding experimental crystal structure as the reference for simulations starting from the closed (red), ajar (green), and open (blue) conformations for the A) dCTP-dG base pair, B) dTTP-dG base pair, and C) dGTP-dG base pair.

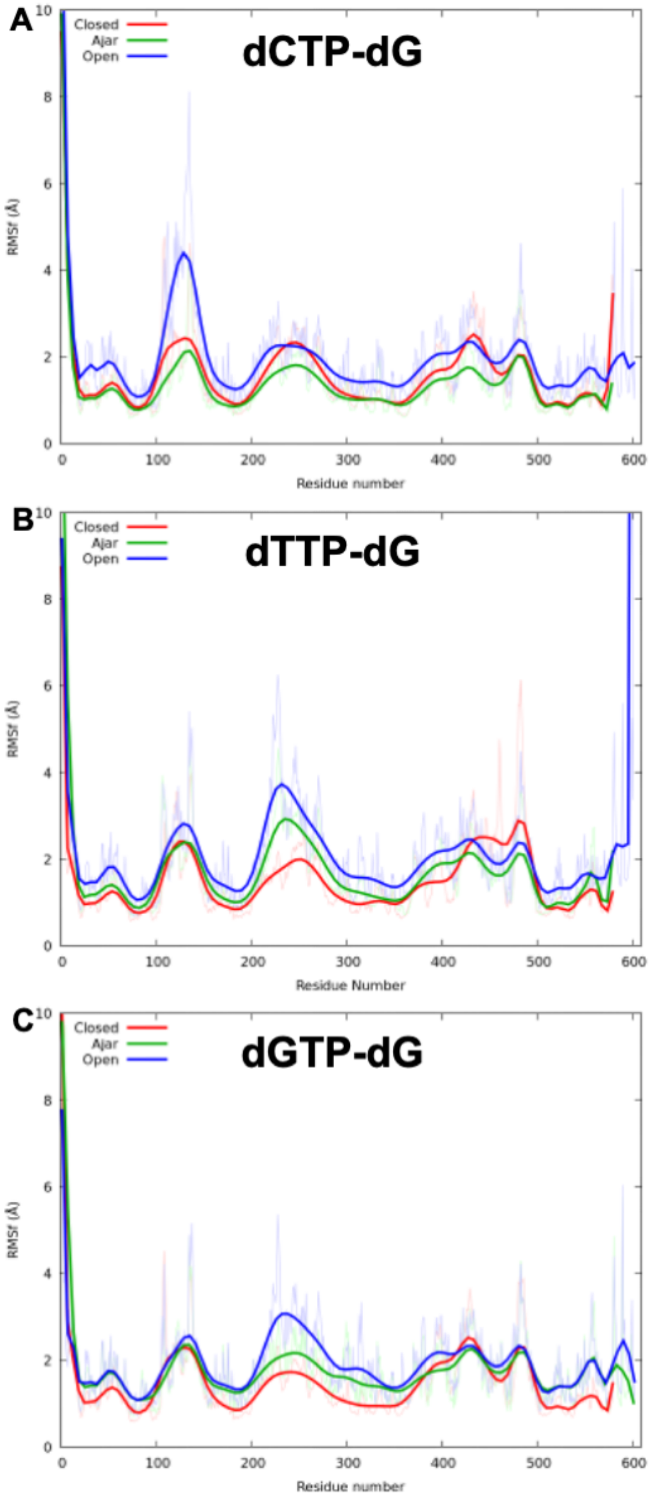

Figure S2. Root mean square fluctuation (RMSf) plots for for each residue for simulations starting from the closed (red), ajar (green), and open (blue) conformations for the A) dCTP-dG base pair, B) dTTP-dG base pair, and C) dGTP-dG base pair. The DNA polymerase amino acids are numbered 1-580, and the DNA nucleotides are numbered 581-610.

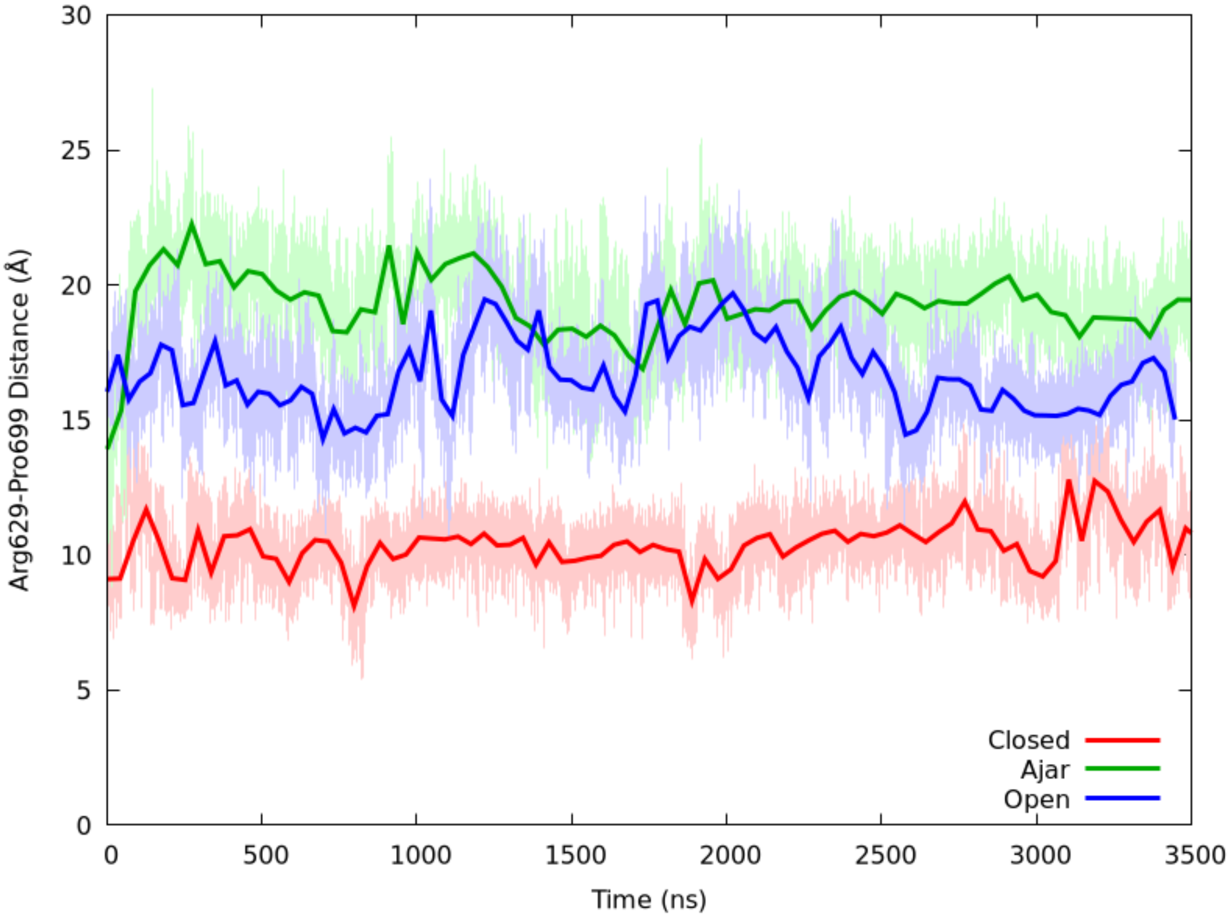

Figure S3. Opening of the fingers domain for the dTTP-dG mismatch simulations initiated from the open (PDB 4YFU, bluegreen), and ajar (PDB 3HPO, greenred), and closed (PDB 1LV5, red) conformations. The opening is measured using the distance between the relatively stationary Arg629 in the palm domain and the more mobile Pro699 found in the O-helix of the fingers domain.

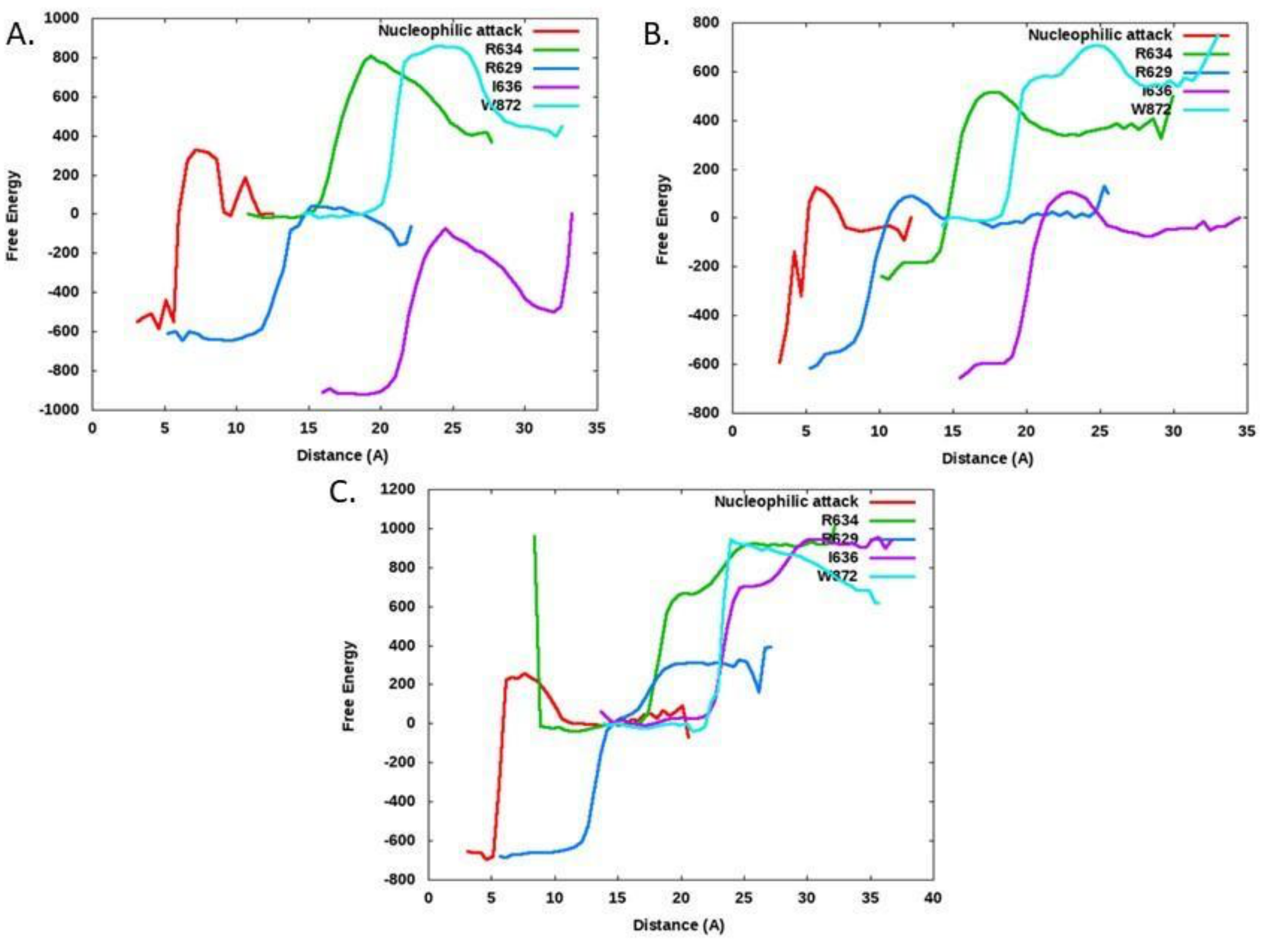

Figure S4. Relative free energy landscape for the prechemistry events for DNA polymerase I in the case of a WC base pair (dCTP-dG, A.), a mismatched non WC base pair(dTTP-dG, B.), and a bad mismatched non-WC base pair in (dGTP-dG, C.). Barriers heights are defined in the direction of polymerase closing (open-to-closed). Free energies are shown in kcal/mol and do not directly include entropic contributions.

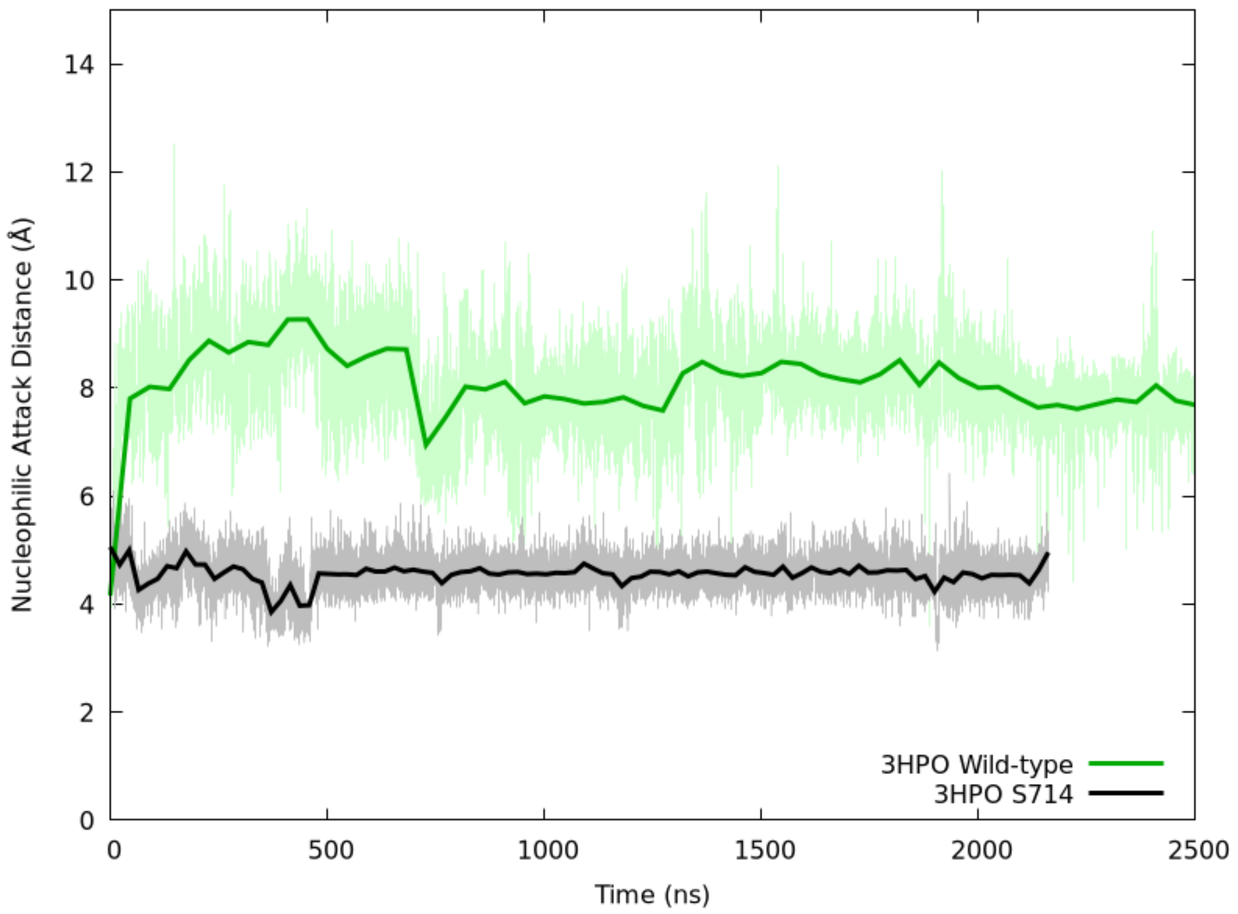

Figure S5. The nucleophilic attack distances (Å) from the 3'-OH of the terminal residue on the growing strand to the α-phosphate of the dNTP in the active site for simulations starting from the ajar (PDB 3HPO) conformation for the wild-type sequence (green) of *B. thermophilus* DNA polymerase I and the Y714S mutation (black).

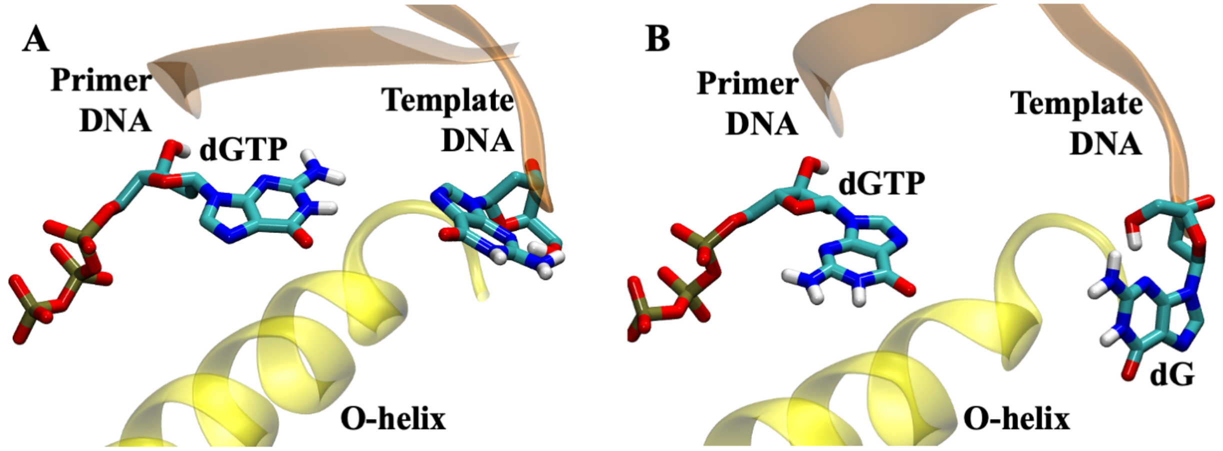

Figure S6. Depiction of the dGTP base changing conformations in the dG-dGTP simulation began from the ajar (3HPO) conformation. A) The original conformation of the dGTP base at the beginning of the simulation. B) The conformation of the dGTP after rotation around the N-glycosidic bond that was observed in the presence of the dG template base.

EXAMPLE INPUT AND OUTPUT FILES

```
%NProcShared=10
#P HF/6-31G*
# Gfinput IOP(6/7=3) iop(6/33=2) iop(6/42=6) iop(6/50=1)  Pop=full  Pop=SaveESP Pop=MK Density
Test SCF=QC
# Units(Ang,Deg)

Input file

-4    1
P     0     -13.260     -0.466      14.089
O     0     -14.504     -0.232      13.263
O     0     -13.001     0.642       15.072
O     0     -12.008     -0.664      13.112
P     0     -10.779     0.354       12.903
O     0     -9.677      -0.453      12.263
O     0     -10.338     1.023       14.158
O     0     -11.324     1.398       11.788
P     0     -11.951     2.860       12.090
O     0     -13.012     3.100       11.042
O     0     -12.505     2.838       13.487
O     0     -10.935     3.957       11.968
O     0     -13.362     -1.866      14.857
C     0     -12.268     -2.222      15.705
C     0     -11.757     -3.614      15.353
O     0     -12.783     -4.609      15.402
C     0     -11.255     -3.747      13.931
O     0     -9.996      -3.111      13.727
C     0     -11.251     -5.250      13.727
C     0     -12.500     -5.669      14.489
N     0     -13.576     -5.679      13.516
C     0     -13.696     -6.778      12.649
O     0     -12.860     -7.715      12.755
N     0     -14.671     -6.812      11.726
C     0     -15.554     -5.796      11.630
O     0     -16.466     -5.813      10.771
C     0     -15.437     -4.632      12.542
C     0     -16.396     -3.467      12.475
C     0     -14.412     -4.636      13.472
H     0     -11.459     -1.503      15.576
H     0     -12.596     -2.216      16.744
H     0     -11.031     -3.935      16.100
H     0     -12.004     -3.356      13.243
H     0     -9.724      -3.224      12.813
H     0     -11.304     -5.473      12.661
H     0     -10.335     -5.671      14.141
H     0     -12.203     -6.558      15.045
H     0     -14.746     -7.601      11.099
H     0     -16.126     -2.730      13.231
H     0     -16.344     -3.009      11.487
H     0     -17.411     -3.820      12.659
H     0     -14.283     -3.811      14.157

TTP.esp
```

Figure S7. Gaussian 09 input file for calculating the partial atomic RESP charges for dTTP.

```
DCP
   40    41     1     0     0
SMALL
resp


@<TRIPOS>ATOM
      1 N1          4.6530   -0.4460   -0.1050 N*        1 DCP     -0.327325
      2 C2          5.8680   -1.0320   -0.5350 C         1 DCP      1.092306
      3 N3          7.0110   -0.6820    0.0590 NC        1 DCP     -1.025947
      4 C4          6.9820    0.2370    1.0800 CA        1 DCP      1.091516
      5 C5          5.8300    0.8430    1.5410 CM        1 DCP     -0.720235
      6 C6          4.6280    0.4940    0.9390 CM        1 DCP      0.247320
      7 N4          8.2620    0.5470    1.6740 N2        1 DCP     -1.107834
      8 O2          5.8120   -1.8600   -1.4440 O         1 DCP     -0.722733
      9 C1'         3.3960   -0.8610   -0.6700 CT        1 DCP     -0.065204
     10 C2'         2.4650   -1.6860    0.2430 CT        1 DCP     -0.068526
     11 C3'         1.0940   -1.2160   -0.1700 CT        1 DCP      0.465954
     12 C4'         1.2980   -0.1870   -1.2560 CT        1 DCP     -0.085302
     13 O4'         2.6240    0.2660   -1.0340 OS        1 DCP     -0.360732
     14 C5'         0.2870    0.9480   -1.3640 CT        1 DCP      0.389378
     15 O5'        -0.1080    1.4360   -0.1190 OS        1 DCP     -0.659121
     16 PA         -1.4680    2.3610   -0.0230 P         1 DCP      1.367244
     17 O1A        -1.3630    3.1290    1.2340 O2        1 DCP     -0.867327
     18 O2A        -1.6290    3.0390   -1.3290 O2        1 DCP     -0.867327
     19 O3A        -2.6270    1.2440    0.1990 OS        1 DCP     -0.573013
     20 PB         -3.3260    0.0150   -0.5920 P         1 DCP      1.508150
     21 O1B        -2.2670   -0.9850   -0.9170 O2        1 DCP     -0.860970
     22 O2B        -4.2340    0.4890   -1.6490 O2        1 DCP     -0.860970
     23 O3B        -4.1970   -0.5620    0.6690 OS        1 DCP     -0.714629
     24 PG         -5.6680   -1.1730    0.8780 P         1 DCP      1.645764
     25 O1G        -5.8670   -1.4000    2.3280 O2        1 DCP     -1.069426
     26 O2G        -6.6680    0.1160    0.5780 O2        1 DCP     -1.069426
     27 O3G        -5.9870   -2.2110   -0.1210 O2        1 DCP     -1.069426
     28 O3'         0.1000   -2.1240   -0.2400 OH        1 DCP     -0.743813
     29 H5          5.8620    1.5690    2.3340 HA        1 DCP      0.229705
     30 H6          3.6820    0.9500    1.1430 H4        1 DCP      0.239640
     31 HN1         8.6590   -0.3360    1.9560 H         1 DCP      0.401161
     32 HN2         8.8620    0.8250    0.9120 H         1 DCP      0.401161
     33 H1'         3.6350   -1.4310   -1.5530 H2        1 DCP      0.187597
     34 H2'1        2.6000   -2.7530    0.0790 HC        1 DCP      0.027464
     35 H2'2        2.6710   -1.4860    1.2930 HC        1 DCP      0.027464
     36 H4'         1.2810   -0.6940   -2.2320 H1        1 DCP      0.078441
     37 H5'1        0.7310    1.7400   -1.9600 H1        1 DCP     -0.017367
     38 H5'2       -0.5620    0.5590   -1.9050 H1        1 DCP     -0.017367
     39 HO         -0.7600   -1.6930   -0.4080 HO        1 DCP      0.440951
     40 H3'         0.9730   -1.1100    0.9020 H1        1 DCP      0.032805
```

Figure S8. Partial atomic charges for dCTP as calculated by Gaussian 09. Details of the Gaussian 09 calculation can be found in Figure S7.

```
@<TRIPOS>MOLECULE
TTP
   42    43     1     0     0
SMALL
resp


@<TRIPOS>ATOM
      1 PA          1.2000    0.3650    1.2580 P         1 TTP      0.940007
      2 O1A         1.0990    1.8710    1.1810 O2        1 TTP     -0.661747
      3 O2A         2.3060   -0.0960    2.1650 O2        1 TTP     -0.661747
      4 O3A         1.3900   -0.2260   -0.2170 OS        1 TTP     -0.542797
      5 PB          2.7180   -0.9100   -0.8170 P         1 TTP      1.542562
      6 O1B         2.2720   -1.6860   -2.0310 O2        1 TTP     -0.902666
      7 O2B         3.4470   -1.7610    0.1650 O2        1 TTP     -0.902666
      8 O3B         3.5990    0.3460   -1.3420 OS        1 TTP     -0.835142
      9 PG          4.8170    1.0470   -0.5370 P         1 TTP      1.698965
     10 O1G         4.7800    2.5150   -0.8920 O2        1 TTP     -1.058675
     11 O2G         4.5990    0.7950    0.9280 O2        1 TTP     -1.058675
     12 O3G         6.1580    0.5140   -0.9460 O2        1 TTP     -1.058675
     13 O5'        -0.1950   -0.2580    1.7350 OS        1 TTP     -0.477153
     14 C5'        -0.2540   -1.6790    1.8820 CT        1 TTP     -0.239856
     15 C4'        -1.4350   -2.2420    1.1010 CT        1 TTP      0.416607
     16 O4'        -2.6800   -1.6400    1.4690 OS        1 TTP     -0.549105
     17 C3'        -1.3800   -1.9690   -0.3870 CT        1 TTP      0.435020
     18 O3'        -0.4110   -2.7680   -1.0600 OH        1 TTP     -0.775823
     19 C2'        -2.8150   -2.2120   -0.8150 CT        1 TTP     -0.080948
     20 C1'        -3.5900   -1.6550    0.3700 CT        1 TTP      0.205272
     21 N1         -3.8730   -0.2690    0.0460 N*        1 TTP     -0.148392
     22 C2         -4.9350    0.0200   -0.8270 C         1 TTP      0.709504
     23 O2         -5.6030   -0.9430   -1.2920 O         1 TTP     -0.712322
     24 N3         -5.2140    1.2900   -1.1600 NA        1 TTP     -0.546163
     25 C4         -4.4850    2.3040   -0.6500 C         1 TTP      0.648899
     26 O4         -4.7320    3.4940   -0.9560 O         1 TTP     -0.673453
     27 C5         -3.3620    2.0100    0.2740 CM        1 TTP     -0.113178
     28 C5M        -2.5120    3.1070    0.8700 CT        1 TTP     -0.096371
     29 C6         -3.1060    0.6860    0.5830 CM        1 TTP     -0.186518
     30 H5''        0.6670   -2.1220    1.5030 H1        1 TTP      0.165004
     31 H5'        -0.3710   -1.9310    2.9350 H1        1 TTP      0.165004
     32 H4'        -1.5620   -3.2970    1.3430 H1        1 TTP     -0.042170
     33 H3'        -1.1950   -0.9090   -0.5550 H1        1 TTP      0.038696
     34 HO3'       -0.4160   -2.5570   -1.9970 HO        1 TTP      0.403031
     35 H2'        -3.0120   -1.6780   -1.7450 HC        1 TTP      0.026563
     36 H2''       -2.9740   -3.2790   -0.9680 HC        1 TTP      0.026563
     37 H1'        -4.3760   -2.3860    0.5590 H2        1 TTP      0.067190
     38 H3         -5.9740    1.4910   -1.7960 H         1 TTP      0.362930
     39 H71        -1.7510    2.6670    1.5150 HC        1 TTP      0.032775
     40 H72        -2.0290    3.6690    0.0700 HC        1 TTP      0.032775
     41 H73        -3.1410    3.7770    1.4560 HC        1 TTP      0.032775
     42 H6         -2.2980    0.4190    1.2490 H4        1 TTP      0.374100
```

Figure S9. Partial atomic charges for dTTP as calculated by Gaussian 09. Details of the Gaussian 09 calculation can be found in Figure S7.

```
GTP
   43    45     1     0     0
SMALL
resp


@<TRIPOS>ATOM
      1  C1'       39.8510    35.0120  -111.9820 CT        1 GTP      0.450579
      2  C2'       40.6610    36.3270  -111.8940 CT        1 GTP     -0.512066
      3  C3'       42.0620    35.8930  -112.2350 CT        1 GTP      0.939283
      4  C4'       41.8220    34.8550  -113.3140 CT        1 GTP      0.252345
      5  O4'       40.6650    34.1040  -112.8240 OS        1 GTP     -0.565462
      6  O3'       42.7830    37.0050  -112.7710 OH        1 GTP     -0.962508
      7  C5'       43.0250    33.9670  -113.6050 CI        1 GTP      0.162416
      8  O5'       43.2950    33.1000  -112.4980 OS        1 GTP     -0.691024
      9  PA        44.7840    32.9990  -111.9010 P         1 GTP      1.105959
     10  O1A       44.8180    31.9560  -110.8670 O2        1 GTP     -0.749973
     11  O2A       45.7670    32.9100  -113.0140 O2        1 GTP     -0.749973
     12  O3A       44.9300    34.4580  -111.1610 OS        1 GTP     -0.432959
     13  PB        45.7830    35.7340  -111.7320 P         1 GTP      1.155819
     14  O1B       44.9060    36.8210  -111.2300 O2        1 GTP     -0.716791
     15  O2B       45.8950    35.3400  -113.1430 O2        1 GTP     -0.716791
     16  O3B       46.9990    35.3940  -110.7300 OS        1 GTP     -0.848950
     17  PG        48.4020    34.8140  -111.2580 P         1 GTP      2.007655
     18  O1G       48.8700    33.7640  -110.3160 O2        1 GTP     -1.143390
     19  O2G       48.2920    34.4790  -112.6910 O2        1 GTP     -1.143390
     20  O3G       49.3620    36.0870  -111.1260 O2        1 GTP     -1.143390
     21  H1'       38.9950    35.3690  -112.5150 H2        1 GTP      0.007866
     22 1H2'       40.6530    36.6540  -110.8750 HC        1 GTP      0.076611
     23 2H2'       40.3180    36.9810  -112.6690 HC        1 GTP      0.076611
     24  H3'       42.6270    35.5240  -111.4040 H1        1 GTP     -0.182692
     25  H4'       41.6470    35.3130  -114.2650 H1        1 GTP     -0.089475
     26  H3'       42.2360    37.7930  -112.7250 HO        1 GTP      0.458508
     27 1H5'       43.8690    34.6180  -113.6940 H1        1 GTP      0.097472
     28 2H5'       42.7470    33.3370  -114.4240 H1        1 GTP      0.097472
     29  N9        39.2270    35.2440  -111.0940 N*        1 GTP     -0.054406
     30  C8        40.0950    34.5580  -110.2750 CK        1 GTP      0.389952
     31  C4        38.0380    35.3390  -110.4050 CB        1 GTP      0.114947
     32  N7        39.5580    34.2140  -109.1350 NB        1 GTP     -0.639851
     33  H8        41.1040    34.3290  -110.5450 H5        1 GTP      0.132894
     34  C5        38.2600    34.7010  -109.2020 CB        1 GTP      0.034117
     35  N3        36.9080    35.9380  -110.8450 NC        1 GTP     -0.630274
     36  C6        37.2010    34.6330  -108.2570 C         1 GTP      0.768284
     37  C2        35.9330    35.8670  -109.9550 CA        1 GTP      1.015809
     38  O6        37.2000    34.1120  -107.1340 O         1 GTP     -0.664553
     39  N1        36.0510    35.2560  -108.7320 NA        1 GTP     -0.834171
     40  H1        35.2860    35.2240  -108.0880 H         1 GTP      0.365851
     41  N2        34.7430    36.4110  -110.2280 N2        1 GTP     -1.151000
     42  H21       34.1280    36.2790  -109.4490 H         1 GTP      0.456319
     43  H22       34.3870    36.8950  -111.0280 H         1 GTP      0.456319
```

Figure S10. Partial atomic charges for dGTP as calculated by Gaussian 09. Details of the Gaussian 09 calculation can be found in Figure S7.

```
TTP=loadmol2 TTP.mol2
#loadamberparams TTP.frcmod
source leaprc.ff12SB
loadamberparams frcmod.ionsjc_tip3p
loadamberprep magnesium.prep
loadamberparams magnesium.dat
a=loadpdb 3HPO.pdb
saveamberparm a 3HPO.prmtop 3HPO.inpcrd
addIons a Na+ 0
saveamberparm a 3HPO_ions.prmtop 3HPO_ions.inpcrd
solvateOct a TIP3PBOX 12.0
saveamberparm a 3HPO_solv.prmtop 3HPO_solv.inpcrd
```

Figure S11. Example Amber *tleap* input file used to generate the initial Amber parameter and coordinate files used for molecular dynamics simulations.

```
Minimization to relax initial bad contacts, explicit solvent
 &cntrl
   imin=1,
   ncyc=1000,
   maxcyc=5000,
   ntpr=50,
   cut=8,
   iwrap=1,
   ntr=1,
   restraint_wt=10.0,
   restraintmask='!@H=',
 /
```

Figure S12. Example Amber minimization input file used for the minimization steps for molecular dynamics.

```
Explicit solvent initial heating mdin
 &cntrl
   imin=0, irest=0, ntx=1,
   ntpr=50000, ntwx=50000, ntwr=50000, nstlim=1000000,
   dt=0.002, ntt=3, gamma_ln=5.0, ig=-1,
   ntc=2, ntf=2, cut=8, ntb=2, ntp=1,
   iwrap=1, ioutfm=1, nmropt=1,
   ntr=1, restraint_wt=10, restraintmask=':1-601'
 /
 &wt
   TYPE='TEMP0', ISTEP1=0, ISTEP2=100000,
   VALUE1=10.0, VALUE2=100.0,
 /
 &wt
   TYPE='TEMP0', ISTEP1=100001, ISTEP2=500000,
   VALUE1=100.0, VALUE2=335.0,
 /
 &wt TYPE='END' /
```

Figure S13. Example Amber heating input file used to heat systems from 0 to 335 K in 2.0 ns.

```
Explicit solvent molecular dynamics constant pressure MD
 &cntrl
   imin=0, irest=0, ntx=1,
   ntpr=10000, ntwx=10000, ntwr=10000, nstlim=250000,
   dt=0.002, ntt=3, tempi=335,
   temp0=335, gamma_ln=1.0, ig=-1,
   ntp=1, ntc=2, ntf=2, cut=9,
   ntb=2, iwrap=1, ioutfm=1,
   ntr=1,
 /
Fix heavy atoms
10.0
FIND
* N3 * *
* CT * *
* C * *
* O * *
* N * *
* OH * *
* N2 * *
* CA * *
* O2 * *
SEARCH
RES 1 601
END
Fix heavy atoms
10.0
FIND
* CC * *
* NB * *
* CR * *
* NA * *
* CW * *
* S * *
* C* * *
* CN * *
* CB * *
* SH * *
SEARCH
RES 1 601
END
END
```

Figure S14. Example Amber equilibration input file used to equilibrate systems prior to unrestrained production molecular dynamics.

```
Explicit solvent molecular dynamics constant pressure 200 ns MD
 &cntrl
   imin=0, irest=1, ntx=5,
   ntpr=50000, ntwx=50000, ntwr=50000, nstlim=100000000,
   dt=0.002, ntt=3, tempi=335,
   temp0=335, gamma_ln=1.0, ig=-1,
   ntp=1, ntc=2, ntf=2, cut=9,
   ntb=2, iwrap=1, ioutfm=1,
 /
```

Figure S15. Example Amber equilibration input file used to perform constant temperature (335 K) unrestrained production MD.

```
MMPBSA.py input file
&general
  startframe=2, endframe=9999999999, interval=1,
  keep_files=1, netcdf=1,
  full_traj=1, strip_mask=:Na+:WAT,
/
&gb
 igb=2,
/
&decomp
 idecomp=1,
/
```

Figure S16. Example *MMPBSA.py* input used to calculate MM-GBSA free energies.
